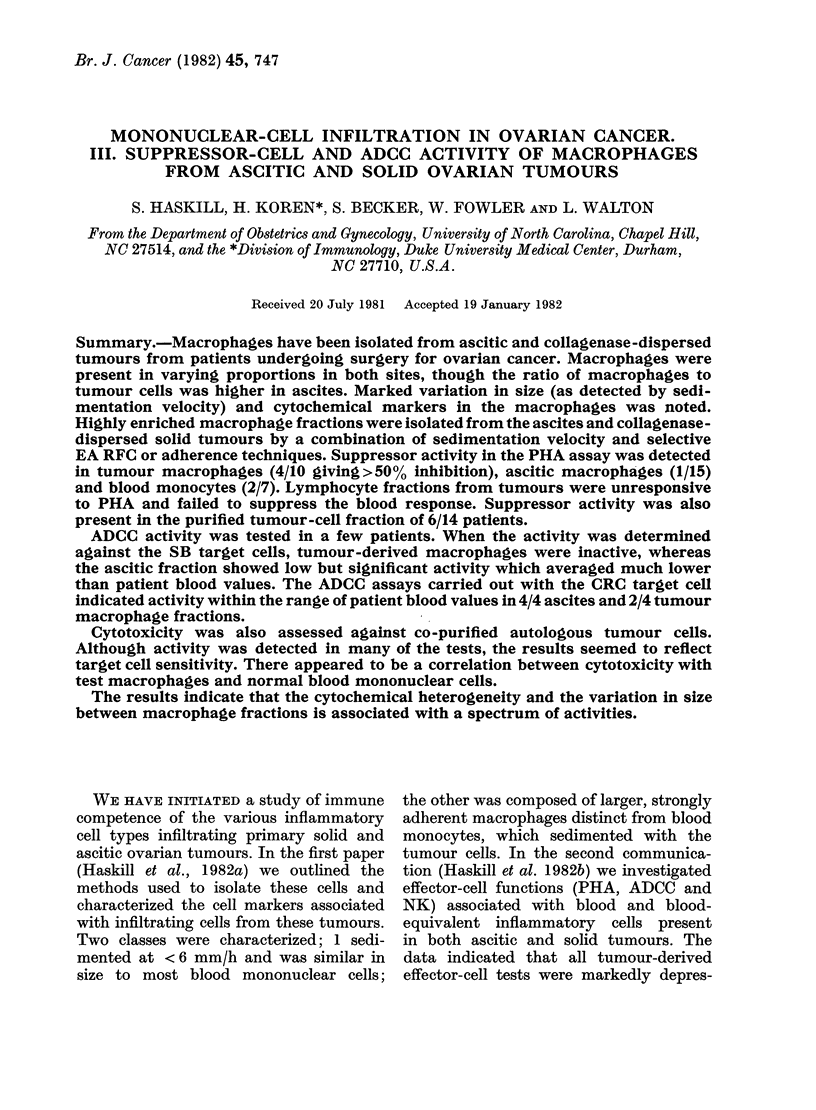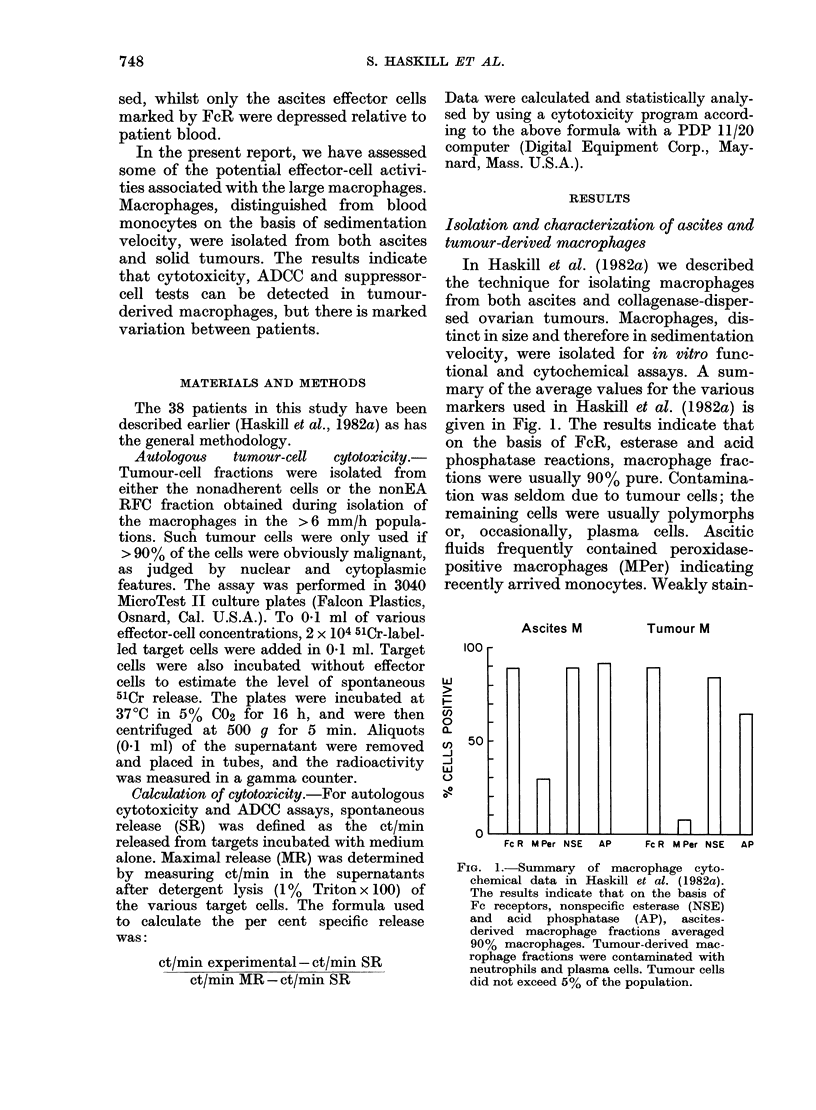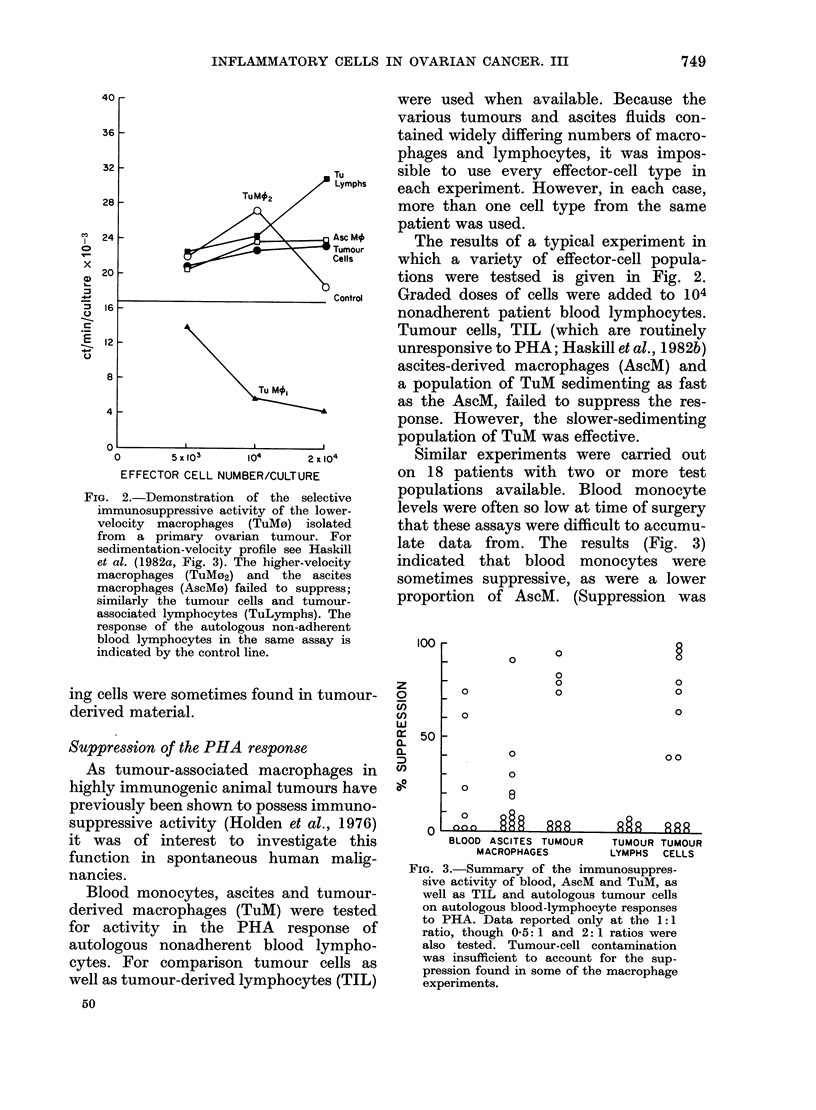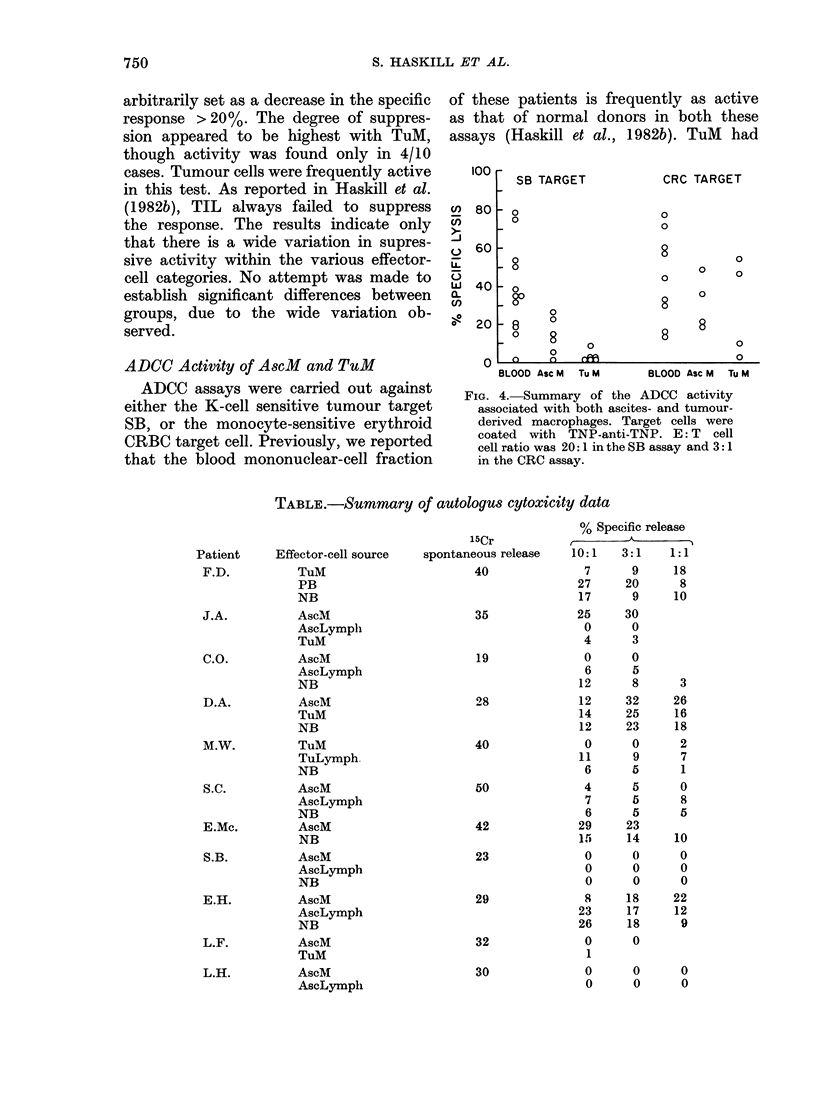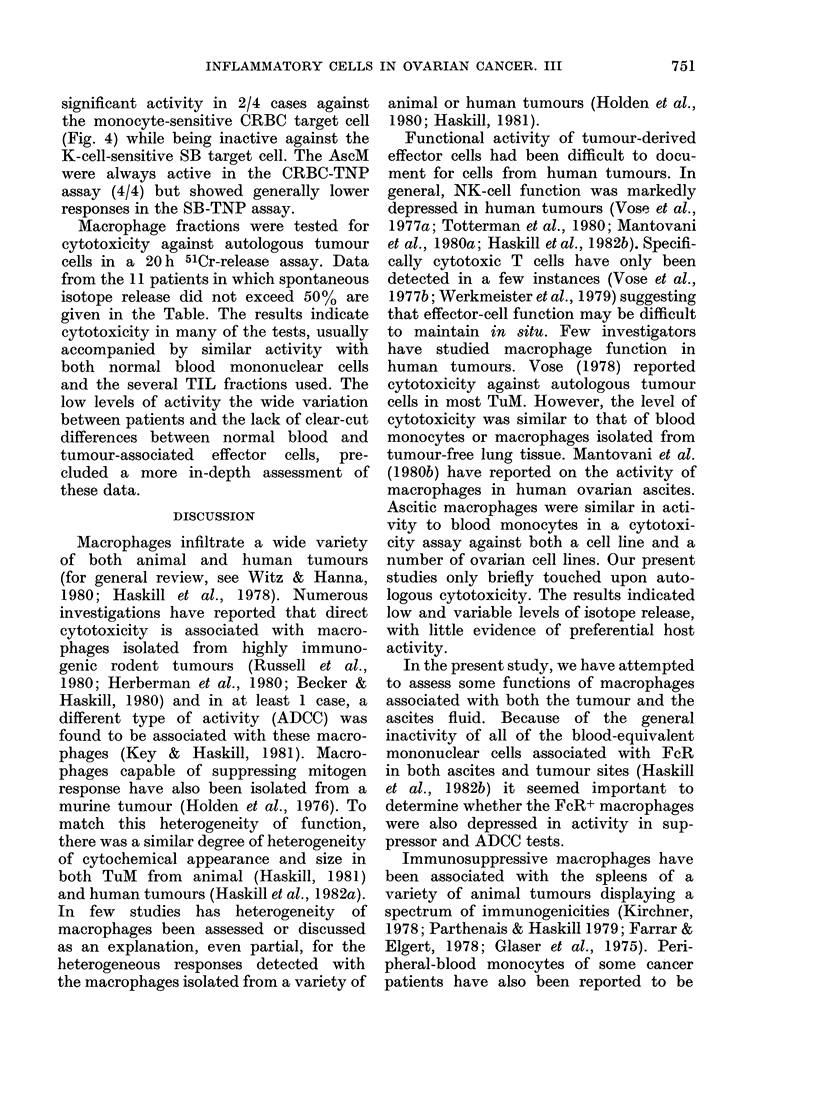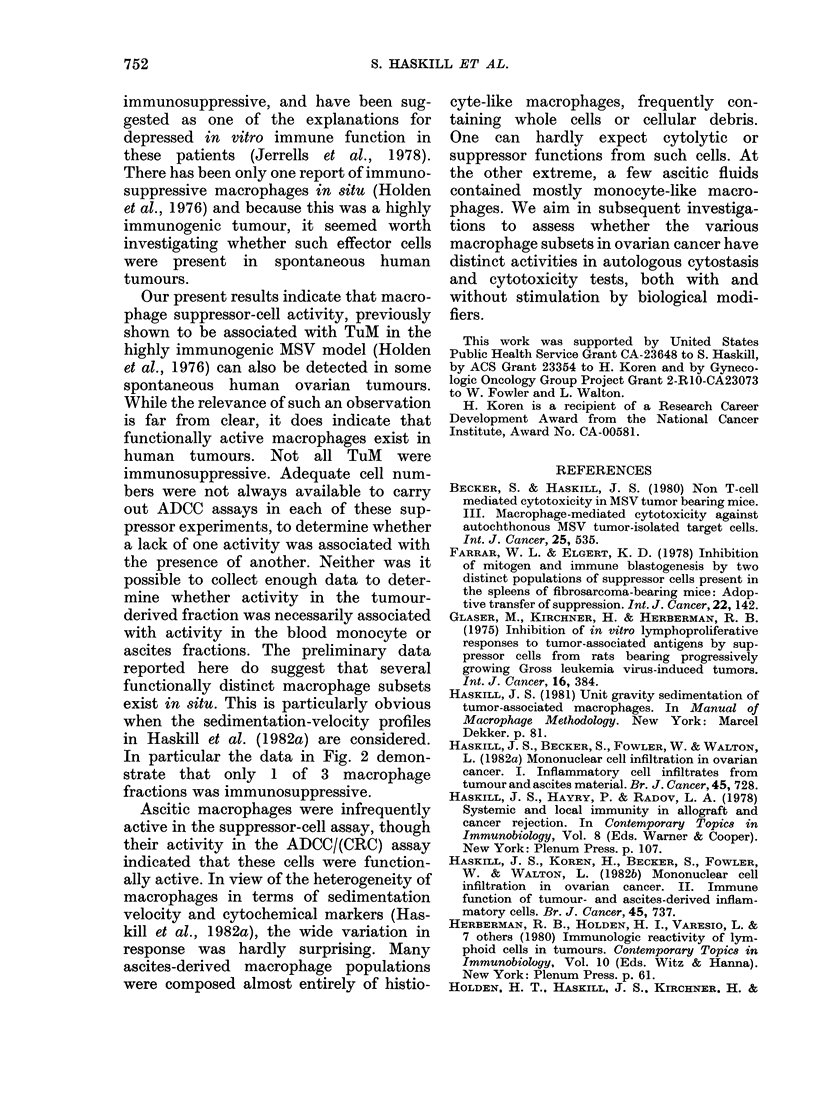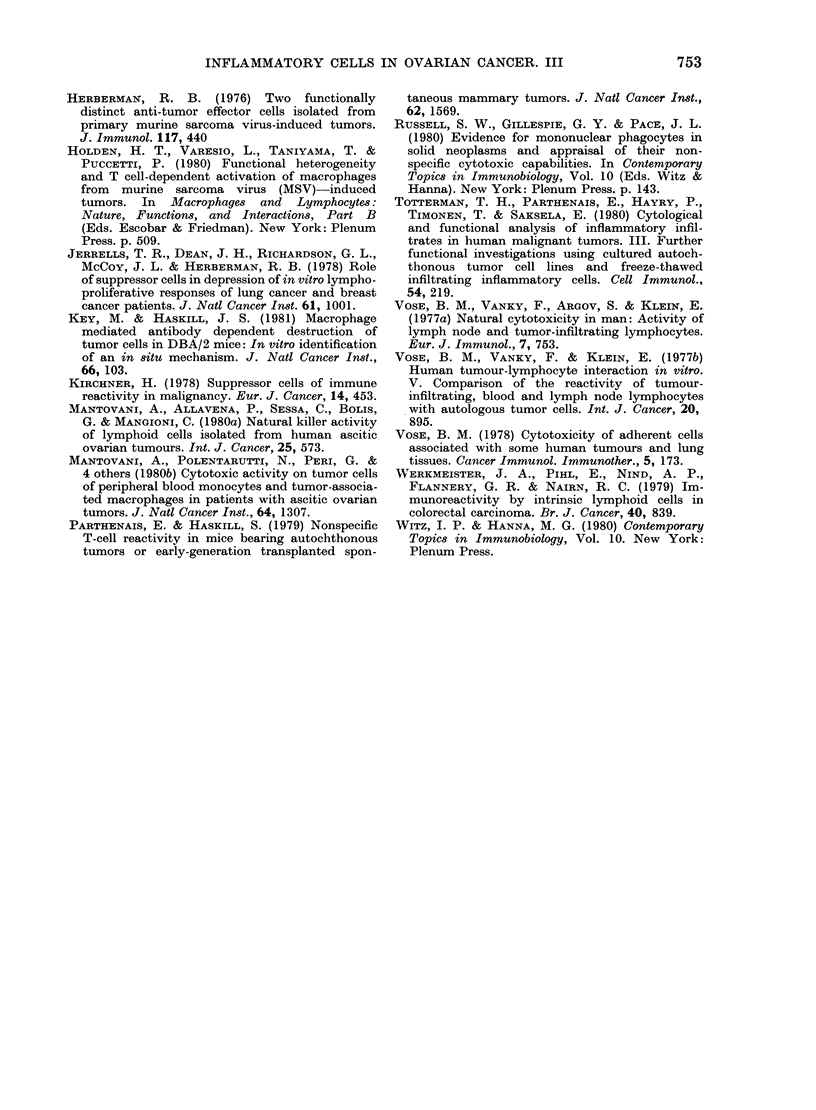# Mononuclear-cell infiltration in ovarian cancer. III. Suppressor-cell and ADCC activity of macrophages from ascitic and solid ovarian tumours.

**DOI:** 10.1038/bjc.1982.116

**Published:** 1982-05

**Authors:** S. Haskill, H. Koren, S. Becker, W. Fowler, L. Walton

## Abstract

Macrophages have been isolated from ascitic and collagenase-dispersed tumours from patients undergoing surgery for ovarian cancer. Macrophages were present in varying proportions in both sites, though the ration of macrophages to tumour cells was higher in ascites. Marked variation in size (as detected by sedimentation velocity) and cytochemical markers in the macrophages was noted. Highly enriched macrophage fractions were isolated from the ascites and collagenase-dispersed solid tumours by a combination of sedimentation velocity and selective EA RFC or adherence techniques. Suppressor activity in the PHA assay was detected in tumour macrophages (4/10 giving less than 50% inhibition), ascitic macrophages (1/15) and blood monocytes (2/7). Lymphocyte fractions from tumours were unresponsive to PHA and failed to suppress the blood response. Suppressor activity was also present in the purified tumour-cell fraction of 6/14 patients. ADCC activity was tested in a few patients. When the activity was determined against the SB target cells, tumour-derived macrophages were inactive, whereas the ascitic fraction showed low but significant activity which averaged much lower than patients blood values. The ADCC assays carried out with the CRC target cell indicated activity within the range of patient blood values in 4/4 ascites and 2/4 tumour macrophage fractions. Cytotoxicity was also assessed against co-purified autologous tumour cells. Although activity was detected in many of the tests, the results seemed to reflect target cell sensitivity. There appeared to be a correlation between cytotoxicity with test macrophages and normal blood mononuclear cells. The results indicate that the cytochemical heterogeneity and the variation in size between macrophage fractions is associated with a spectrum of activities.


Br. J. Cancer (1982) 45, 747

### MONONUCLEAR-CELL INFILTRATION IN OVARIAN CANCER. III. SUPPRESSOR-CELL AND ADCC ACTIVITY OF MACROPHAGES FROM ASCITIC AND SOLID OVARIAN TUMOURS

S. HASKILL, H. KOREN*, S. BECKER, W. FOWLER AND L. WALTON

*From the Department of Obstetrics and Gynecology, University of North Carolina, Chapel Hill, NC 27514, and the *Division of Immunology, Duke University Medical Center, Durham, NC 27710, U.S.A.*

Received 20 July 1981 Accepted 19 January 1982

**Summary.—Macrophages have been isolated from ascitic and collagenase-dispersed tumours from patients undergoing surgery for ovarian cancer. Macrophages were present in varying proportions in both sites, though the ratio of macrophages to tumour cells was higher in ascites. Marked variation in size (as detected by sedimentation velocity) and cytochemical markers in the macrophages was noted. Highly enriched macrophage fractions were isolated from the ascites and collagenase-dispersed solid tumours by a combination of sedimentation velocity and selective EA RFC or adherence techniques. Suppressor activity in the PHA assay was detected in tumour macrophages (4/10 giving >50% inhibition), ascitic macrophages (1/15) and blood monocytes (2/7). Lymphocyte fractions from tumours were unresponsive to PHA and failed to suppress the blood response. Suppressor activity was also present in the purified tumour-cell fraction of 6/14 patients.**

**ADCC activity was tested in a few patients. When the activity was determined against the SB target cells, tumour-derived macrophages were inactive, whereas the ascitic fraction showed low but significant activity which averaged much lower than patient blood values. The ADCC assays carried out with the CRC target cell indicated activity within the range of patient blood values in 4/4 ascites and 2/4 tumour macrophage fractions.**

**Cytotoxicity was also assessed against co-purified autologous tumour cells. Although activity was detected in many of the tests, the results seemed to reflect target cell sensitivity. There appeared to be a correlation between cytotoxicity with test macrophages and normal blood mononuclear cells.**

**The results indicate that the cytochemical heterogeneity and the variation in size between macrophage fractions is associated with a spectrum of activities.**

WE HAVE INITIATED a study of immune competence of the various inflammatory cell types infiltrating primary solid and ascitic ovarian tumours. In the first paper (Haskill *et al.*, 1982*a*) we outlined the methods used to isolate these cells and characterized the cell markers associated with infiltrating cells from these tumours. Two classes were characterized; 1 sedimented at < 6 mm/h and was similar in size to most blood mononuclear cells; the other was composed of larger, strongly adherent macrophages distinct from blood monocytes, which sedimented with the tumour cells. In the second communication (Haskill *et al.* 1982*b*) we investigated effector-cell functions (PHA, ADCC and NK) associated with blood and blood-equivalent inflammatory cells present in both ascitic and solid tumours. The data indicated that all tumour-derived effector-cell tests were markedly depressed, whilst only the ascites effector cells marked by FcR were depressed relative to patient blood.

In the present report, we have assessed some of the potential effector-cell activities associated with the large macrophages. Macrophages, distinguished from blood monocytes on the basis of sedimentation velocity, were isolated from both ascites and solid tumours. The results indicate that cytotoxicity, ADCC and suppressor-cell tests can be detected in tumour-derived macrophages, but there is marked variation between patients.

### MATERIALS AND METHODS

The 38 patients in this study have been described earlier (Haskill *et al.*, 1982*a*) as has the general methodology.

*Autologous tumour-cell cytotoxicity.*—Tumour-cell fractions were isolated from either the nonadherent cells or the nonEA RFC fraction obtained during isolation of the macrophages in the $>6$ mm/h populations. Such tumour cells were only used if $>90\%$ of the cells were obviously malignant, as judged by nuclear and cytoplasmic features. The assay was performed in 3040 MicroTest II culture plates (Falcon Plastics, Osnard, Cal. U.S.A.). To 0·1 ml of various effector-cell concentrations, $2 \times 10^4$ $^{51}Cr$-labelled target cells were added in 0·1 ml. Target cells were also incubated without effector cells to estimate the level of spontaneous $^{51}Cr$ release. The plates were incubated at 37°C in 5% $CO_2$ for 16 h, and were then centrifuged at 500 *g* for 5 min. Aliquots (0·1 ml) of the supernatant were removed and placed in tubes, and the radioactivity was measured in a gamma counter.

*Calculation of cytotoxicity.*—For autologous cytotoxicity and ADCC assays, spontaneous release (SR) was defined as the ct/min released from targets incubated with medium alone. Maximal release (MR) was determined by measuring ct/min in the supernatants after detergent lysis (1% Triton $\times$ 100) of the various target cells. The formula used to calculate the per cent specific release was:

$$\frac{\text{ct/min experimental} - \text{ct/min SR}}{\text{ct/min MR} - \text{ct/min SR}}$$

Data were calculated and statistically analysed by using a cytotoxicity program according to the above formula with a PDP 11/20 computer (Digital Equipment Corp., Maynard, Mass. U.S.A.).

### RESULTS

### *Isolation and characterization of ascites and tumour-derived macrophages*

In Haskill *et al.* (1982*a*) we described the technique for isolating macrophages from both ascites and collagenase-dispersed ovarian tumours. Macrophages, distinct in size and therefore in sedimentation velocity, were isolated for *in vitro* functional and cytochemical assays. A summary of the average values for the various markers used in Haskill *et al.* (1982*a*) is given in Fig. 1. The results indicate that on the basis of FcR, esterase and acid phosphatase reactions, macrophage fractions were usually 90% pure. Contamination was seldom due to tumour cells; the remaining cells were usually polymorphs or, occasionally, plasma cells. Ascitic fluids frequently contained peroxidase-positive macrophages (MPer) indicating recently arrived monocytes. Weakly stain-

FIG. 1.—Summary of macrophage cytochemical data in Haskill *et al.* (1982*a*). The results indicate that on the basis of Fc receptors, nonspecific esterase (NSE) and acid phosphatase (AP), ascites-derived macrophage fractions averaged 90% macrophages. Tumour-derived macrophage fractions were contaminated with neutrophils and plasma cells. Tumour cells did not exceed 5% of the population.

FIG. 2.—Demonstration of the selective immunosuppressive activity of the lower-velocity macrophages (TuMø) isolated from a primary ovarian tumour. For sedimentation-velocity profile see Haskill *et al.* (1982*a*, Fig. 3). The higher-velocity macrophages ($TuMø_2$) and the ascites macrophages (AscMø) failed to suppress; similarly the tumour cells and tumour-associated lymphocytes (TuLymphs). The response of the autologous non-adherent blood lymphocytes in the same assay is indicated by the control line.

ing cells were sometimes found in tumour-derived material.

### *Suppression of the PHA response*

As tumour-associated macrophages in highly immunogenic animal tumours have previously been shown to possess immunosuppressive activity (Holden *et al.*, 1976) it was of interest to investigate this function in spontaneous human malignancies.

Blood monocytes, ascites and tumour-derived macrophages (TuM) were tested for activity in the PHA response of autologous nonadherent blood lymphocytes. For comparison tumour cells as well as tumour-derived lymphocytes (TIL) were used when available. Because the various tumours and ascites fluids contained widely differing numbers of macrophages and lymphocytes, it was impossible to use every effector-cell type in each experiment. However, in each case, more than one cell type from the same patient was used.

The results of a typical experiment in which a variety of effector-cell populations were testsed is given in Fig. 2. Graded doses of cells were added to $10^4$ nonadherent patient blood lymphocytes. Tumour cells, TIL (which are routinely unresponsive to PHA; Haskill *et al.*, 1982*b*) ascites-derived macrophages (AscM) and a population of TuM sedimenting as fast as the AscM, failed to suppress the response. However, the slower-sedimenting population of TuM was effective.

Similar experiments were carried out on 18 patients with two or more test populations available. Blood monocyte levels were often so low at time of surgery that these assays were difficult to accumulate data from. The results (Fig. 3) indicated that blood monocytes were sometimes suppressive, as were a lower proportion of AscM. (Suppression was

FIG. 3.—Summary of the immunosuppressive activity of blood, AscM and TuM, as well as TIL and autologous tumour cells on autologous blood-lymphocyte responses to PHA. Data reported only at the 1:1 ratio, though 0·5:1 and 2:1 ratios were also tested. Tumour-cell contamination was insufficient to account for the suppression found in some of the macrophage experiments.

arbitrarily set as a decrease in the specific response >20%. The degree of suppression appeared to be highest with TuM, though activity was found only in 4/10 cases. Tumour cells were frequently active in this test. As reported in Haskill *et al.* (1982*b*), TIL always failed to suppress the response. The results indicate only that there is a wide variation in supressive activity within the various effector-cell categories. No attempt was made to establish significant differences between groups, due to the wide variation observed.

### *ADCC Activity of AscM and TuM*

ADCC assays were carried out against either the K-cell sensitive tumour target SB, or the monocyte-sensitive erythroid CRBC target cell. Previously, we reported that the blood mononuclear-cell fraction of these patients is frequently as active as that of normal donors in both these assays (Haskill *et al.*, 1982*b*). TuM had

FIG. 4.—Summary of the ADCC activity associated with both ascites- and tumour-derived macrophages. Target cells were coated with TNP-anti-TNP. E:T cell cell ratio was 20:1 in the SB assay and 3:1 in the CRC assay.

TABLE.—*Summary of autologus cytoxicity data*

| Patient | Effector-cell source | $^{15}$Cr spontaneous release | % Specific release 10:1 | 3:1 | 1:1 |
|---|---|---|---|---|---|
| F.D. | TuM | 40 | 7 | 9 | 18 |
| | PB | | 27 | 20 | 8 |
| | NB | | 17 | 9 | 10 |
| J.A. | AscM | 35 | 25 | 30 | |
| | AscLymph | | 0 | 0 | |
| | TuM | | 4 | 3 | |
| C.O. | AscM | 19 | 0 | 0 | |
| | AscLymph | | 6 | 5 | |
| | NB | | 12 | 8 | 3 |
| D.A. | AscM | 28 | 12 | 32 | 26 |
| | TuM | | 14 | 25 | 16 |
| | NB | | 12 | 23 | 18 |
| M.W. | TuM | 40 | 0 | 0 | 2 |
| | TuLymph | | 11 | 9 | 7 |
| | NB | | 6 | 5 | 1 |
| S.C. | AscM | 50 | 4 | 5 | 0 |
| | AscLymph | | 7 | 5 | 8 |
| | NB | | 6 | 5 | 5 |
| E.Mc. | AscM | 42 | 29 | 23 | |
| | NB | | 15 | 14 | 10 |
| S.B. | AscM | 23 | 0 | 0 | 0 |
| | AscLymph | | 0 | 0 | 0 |
| | NB | | 0 | 0 | 0 |
| E.H. | AscM | 29 | 8 | 18 | 22 |
| | AscLymph | | 23 | 17 | 12 |
| | NB | | 26 | 18 | 9 |
| L.F. | AscM | 32 | 0 | 0 | |
| | TuM | | 1 | | |
| L.H. | AscM | 30 | 0 | 0 | 0 |
| | AscLymph | | 0 | 0 | 0 |

significant activity in 2/4 cases against the monocyte-sensitive CRBC target cell (Fig. 4) while being inactive against the K-cell-sensitive SB target cell. The AscM were always active in the CRBC-TNP assay (4/4) but showed generally lower responses in the SB-TNP assay.

Macrophage fractions were tested for cytotoxicity against autologous tumour cells in a 20 h $^{51}$Cr-release assay. Data from the 11 patients in which spontaneous isotope release did not exceed 50% are given in the Table. The results indicate cytotoxicity in many of the tests, usually accompanied by similar activity with both normal blood mononuclear cells and the several TIL fractions used. The low levels of activity the wide variation between patients and the lack of clear-cut differences between normal blood and tumour-associated effector cells, precluded a more in-depth assessment of these data.

### DISCUSSION

Macrophages infiltrate a wide variety of both animal and human tumours (for general review, see Witz & Hanna, 1980; Haskill *et al.*, 1978). Numerous investigations have reported that direct cytotoxicity is associated with macrophages isolated from highly immunogenic rodent tumours (Russell *et al.*, 1980; Herberman *et al.*, 1980; Becker & Haskill, 1980) and in at least 1 case, a different type of activity (ADCC) was found to be associated with these macrophages (Key & Haskill, 1981). Macrophages capable of suppressing mitogen response have also been isolated from a murine tumour (Holden *et al.*, 1976). To match this heterogeneity of function, there was a similar degree of heterogeneity of cytochemical appearance and size in both TuM from animal (Haskill, 1981) and human tumours (Haskill *et al.*, 1982*a*). In few studies has heterogeneity of macrophages been assessed or discussed as an explanation, even partial, for the heterogeneous responses detected with the macrophages isolated from a variety of animal or human tumours (Holden *et al.*, 1980; Haskill, 1981).

Functional activity of tumour-derived effector cells had been difficult to document for cells from human tumours. In general, NK-cell function was markedly depressed in human tumours (Vose *et al.*, 1977*a*; Totterman *et al.*, 1980; Mantovani *et al.*, 1980*a*; Haskill *et al.*, 1982*b*). Specifically cytotoxic T cells have only been detected in a few instances (Vose *et al.*, 1977*b*; Werkmeister *et al.*, 1979) suggesting that effector-cell function may be difficult to maintain *in situ*. Few investigators have studied macrophage function in human tumours. Vose (1978) reported cytotoxicity against autologous tumour cells in most TuM. However, the level of cytotoxicity was similar to that of blood monocytes or macrophages isolated from tumour-free lung tissue. Mantovani *et al.* (1980*b*) have reported on the activity of macrophages in human ovarian ascites. Ascitic macrophages were similar in activity to blood monocytes in a cytotoxicity assay against both a cell line and a number of ovarian cell lines. Our present studies only briefly touched upon autologous cytotoxicity. The results indicated low and variable levels of isotope release, with little evidence of preferential host activity.

In the present study, we have attempted to assess some functions of macrophages associated with both the tumour and the ascites fluid. Because of the general inactivity of all of the blood-equivalent mononuclear cells associated with FcR in both ascites and tumour sites (Haskill *et al.*, 1982*b*) it seemed important to determine whether the FcR$^+$ macrophages were also depressed in activity in suppressor and ADCC tests.

Immunosuppressive macrophages have been associated with the spleens of a variety of animal tumours displaying a spectrum of immunogenicities (Kirchner, 1978; Parthenais & Haskill 1979; Farrar & Elgert, 1978; Glaser *et al.*, 1975). Peripheral-blood monocytes of some cancer patients have also been reported to be immunosuppressive, and have been suggested as one of the explanations for depressed *in vitro* immune function in these patients (Jerrells *et al.*, 1978). There has been only one report of immunosuppressive macrophages *in situ* (Holden *et al.*, 1976) and because this was a highly immunogenic tumour, it seemed worth investigating whether such effector cells were present in spontaneous human tumours.

Our present results indicate that macrophage suppressor-cell activity, previously shown to be associated with TuM in the highly immunogenic MSV model (Holden *et al.*, 1976) can also be detected in some spontaneous human ovarian tumours. While the relevance of such an observation is far from clear, it does indicate that functionally active macrophages exist in human tumours. Not all TuM were immunosuppressive. Adequate cell numbers were not always available to carry out ADCC assays in each of these suppressor experiments, to determine whether a lack of one activity was associated with the presence of another. Neither was it possible to collect enough data to determine whether activity in the tumour-derived fraction was necessarily associated with activity in the blood monocyte or ascites fractions. The preliminary data reported here do suggest that several functionally distinct macrophage subsets exist *in situ*. This is particularly obvious when the sedimentation-velocity profiles in Haskill *et al.* (1982*a*) are considered. In particular the data in Fig. 2 demonstrate that only 1 of 3 macrophage fractions was immunosuppressive.

Ascitic macrophages were infrequently active in the suppressor-cell assay, though their activity in the ADCC/(CRC) assay indicated that these cells were functionally active. In view of the heterogeneity of macrophages in terms of sedimentation velocity and cytochemical markers (Haskill *et al.*, 1982*a*), the wide variation in response was hardly surprising. Many ascites-derived macrophage populations were composed almost entirely of histiocyte-like macrophages, frequently containing whole cells or cellular debris. One can hardly expect cytolytic or suppressor functions from such cells. At the other extreme, a few ascitic fluids contained mostly monocyte-like macrophages. We aim in subsequent investigations to assess whether the various macrophage subsets in ovarian cancer have distinct activities in autologous cytostasis and cytotoxicity tests, both with and without stimulation by biological modifiers.

This work was supported by United States Public Health Service Grant CA-23648 to S. Haskill, by ACS Grant 23354 to H. Koren and by Gynecologic Oncology Group Project Grant 2-R10-CA23073 to W. Fowler and L. Walton.

H. Koren is a recipient of a Research Career Development Award from the National Cancer Institute, Award No. CA-00581.

### REFERENCES

Becker, S. & Haskill, J. S. (1980) Non T-cell mediated cytotoxicity in MSV tumor bearing mice. III. Macrophage-mediated cytotoxicity against autochthonous MSV tumor-isolated target cells. *Int. J. Cancer*, **25**, 535.

Farrar, W. L. & Elgert, K. D. (1978) Inhibition of mitogen and immune blastogenesis by two distinct populations of suppressor cells present in the spleens of fibrosarcoma-bearing mice: Adoptive transfer of suppression. *Int. J. Cancer*, **22**, 142.

Glaser, M., Kirchner, H. & Herberman, R. B. (1975) Inhibition of *in vitro* lymphoproliferative responses to tumor-associated antigens by suppressor cells from rats bearing progressively growing Gross leukemia virus-induced tumors. *Int. J. Cancer*, **16**, 384.

Haskill, J. S. (1981) Unit gravity sedimentation of tumor-associated macrophages. In *Manual of Macrophage Methodology*. New York: Marcel Dekker. p. 81.

Haskill, J. S., Becker, S., Fowler, W. & Walton, L. (1982*a*) Mononuclear cell infiltration in ovarian cancer. I. Inflammatory cell infiltrates from tumour and ascites material. *Br. J. Cancer*, **45**, 728.

Haskill, J. S., Hayry, P. & Radov, L. A. (1978) Systemic and local immunity in allograft and cancer rejection. In *Contemporary Topics in Immunobiology*, Vol. 8 (Eds. Warner & Cooper). New York: Plenum Press. p. 107.

Haskill, J. S., Koren, H., Becker, S., Fowler, W. & Walton, L. (1982*b*) Mononuclear cell infiltration in ovarian cancer. II. Immune function of tumour- and ascites-derived inflammatory cells. *Br. J. Cancer*, **45**, 737.

Herberman, R. B., Holden, H. I., Varesio, L. & 7 others (1980) Immunologic reactivity of lymphoid cells in tumours. *Contemporary Topics in Immunobiology*, Vol. 10 (Eds. Witz & Hanna). New York: Plenum Press. p. 61.

Holden, H. T., Haskill, J. S., Kirchner, H. &

Herberman, R. B. (1976) Two functionally distinct anti-tumor effector cells isolated from primary murine sarcoma virus-induced tumors. *J. Immunol.* **117**, 440

Holden, H. T., Varesio, L., Taniyama, T. & Puccetti, P. (1980) Functional heterogeneity and T cell-dependent activation of macrophages from murine sarcoma virus (MSV)—induced tumors. In *Macrophages and Lymphocytes: Nature, Functions, and Interactions, Part B* (Eds. Escobar & Friedman). New York: Plenum Press. p. 509.

Jerrells, T. R., Dean, J. H., Richardson, G. L., McCoy, J. L. & Herberman, R. B. (1978) Role of suppressor cells in depression of *in vitro* lymphoproliferative responses of lung cancer and breast cancer patients. *J. Natl Cancer Inst.* **61**, 1001.

Key, M. & Haskill, J. S. (1981) Macrophage mediated antibody dependent destruction of tumor cells in DBA/2 mice: *In vitro* identification of an *in situ* mechanism. *J. Natl Cancer Inst.*, **66**, 103.

Kirchner, H. (1978) Suppressor cells of immune reactivity in malignancy. *Eur. J. Cancer*, **14**, 453.

Mantovani, A., Allavena, P., Sessa, C., Bolis, G. & Mangioni, C. (1980*a*) Natural killer activity of lymphoid cells isolated from human ascitic ovarian tumours. *Int. J. Cancer*, **25**, 573.

Mantovani, A., Polentarutti, N., Peri, G. & 4 others (1980*b*) Cytotoxic activity on tumor cells of peripheral blood monocytes and tumor-associated macrophages in patients with ascitic ovarian tumors. *J. Natl Cancer Inst.*, **64**, 1307.

Parthenais, E. & Haskill, S. (1979) Nonspecific T-cell reactivity in mice bearing autochthonous tumors or early-generation transplanted spontaneous mammary tumors. *J. Natl Cancer Inst.*, **62**, 1569.

Russell, S. W., Gillespie, G. Y. & Pace, J. L. (1980) Evidence for mononuclear phagocytes in solid neoplasms and appraisal of their non-specific cytotoxic capabilities. In *Contemporary Topics in Immunobiology*, Vol. 10 (Eds. Witz & Hanna). New York: Plenum Press. p. 143.

Totterman, T. H., Parthenais, E., Hayry, P., Timonen, T. & Saksela, E. (1980) Cytological and functional analysis of inflammatory infiltrates in human malignant tumors. III. Further functional investigations using cultured autochthonous tumor cell lines and freeze-thawed infiltrating inflammatory cells. *Cell Immunol.*, **54**, 219.

Vose, B. M., Vanky, F., Argov, S. & Klein, E. (1977*a*) Natural cytotoxicity in man: Activity of lymph node and tumor-infiltrating lymphocytes. *Eur. J. Immunol.*, **7**, 753.

Vose, B. M., Vanky, F. & Klein, E. (1977*b*) Human tumour-lymphocyte interaction *in vitro*. V. Comparison of the reactivity of tumour-infiltrating, blood and lymph node lymphocytes with autologous tumor cells. *Int. J. Cancer*, **20**, 895.

Vose, B. M. (1978) Cytotoxicity of adherent cells associated with some human tumours and lung tissues. *Cancer Immunol. Immunother.*, **5**, 173.

Werkmeister, J. A., Pihl, E., Nind, A. P., Flannery, G. R. & Nairn, R. C. (1979) Immunoreactivity by intrinsic lymphoid cells in colorectal carcinoma. *Br. J. Cancer*, **40**, 839.

Witz, I. P. & Hanna, M. G. (1980) *Contemporary Topics in Immunobiology*, Vol. 10. New York: Plenum Press.